# Living with Fibrosis: From Diagnosis to Future Hope

**DOI:** 10.3389/fphar.2015.00288

**Published:** 2015-12-16

**Authors:** Matthew A. Sleeman, Joseph Parker, Lynne A. Murray

**Affiliations:** ^1^Respiratory, Inflammation and Autoimmunity, MedImmune Ltd.Cambridge, UK; ^2^Clinical Development, MedImmune LLCGaithersburg, MD, USA

**Keywords:** chronic, disease, GWAS (genome-wide association study), patient experiences, lifelong therapies

Fibrosis, the definition for which being the formation of fibrous tissue in an organ or a specific region of the body, can often be a life threatening pathology or condition. Whilst fibrosis is often readily discernable by simple histology, the causative triggers and events that lead to this excess scarring often remain poorly understood by treating physicians, and the scientific community. Even though there have been two recent notable Phase 3 clinical successes (King et al., 2014; Richeldi, 2014), resulting in the approval of pirfenidone and nintedanib for the treatment of idiopathic pulmonary fibrosis (IPF), the availability of treatment options for patients is often modest and often carry with them many side effects.

One of the key challenges faced in understanding fibrosis is that it is a pathological feature of many diseases rather than a distinct disease by itself and therefore it spans multiple organs and areas of the body. Consequently it is treated by many different specialty care physicians, such as pulmonologists for IPF and nephrologists for renal fibrosis, therefore providing a broader scientific base and diversity of opinions on how to treat diseases with significant fibrotic tissue.

To improve our understanding of these diseases, and in our attempts to bring new therapies forward, it is also key that the patients perspective is given due consideration. In preparation of this short commentary, we sought to get a view from individual patients following diagnosis and living with their disease. As researchers, we focus on *in vitro* and *in vivo* models, and often forget the end goal of what our pre-clinical systems are trying to address. Three individuals have provided a personal view of when they were diagnosed with disease and how this impacts their daily life. The diseases each person has been diagnosed with all have significant tissue fibrosis, however they are very disparate conditions: idiopathic pulmonary fibrosis (IPF), systemic sclerosis (SSc), and autoimmune liver cirrhosis. The extent and location of the fibrosis, and whether this pathophysiology is contributing directly, or potentially being beneficial to the overall disease, is different between each disease setting. However, whilst they are distinct diseases, these patients appear to have similar experiences firstly the insidious onset of symptoms and slow progression of their disease, which further delays a patient from presenting for medical attention, secondly the unsuspecting clinical presentation of the disease, thirdly the complex management of the symptoms, and finally the relentless impact on their quality of life. In the case of IPF, the extent and progression of lung fibrosis is known to directly relate to disease progression and survival (Gay et al, 1998). Fibrotic, aberrant collagen deposition directly impacts gas exchange and the increased tissue stiffness alters the physiological capacity of the lung. In scleroderma, the impact of fibrosis is more apparent in that there's an obvious alteration in skin appearance and also any ensuing internal organ fibrotic change manifesting in decreased function (Gabrielli et al., 2009). Fibrosis of the liver can arise following a number of insults including toxins, viral infections and in the case of the patient associated with this article, autoimmunity. It is known that tackling the cause of fibrosis can allow for a spontaneous resolution of liver fibrosis, as observed with anti-viral in hepatitis c-associated liver fibrosis, or alcohol cessation (Pellicoro et al., 2012). However, poor diet can also play a significant impact on liver repair and fibrosis, and is emerging as a potential future major cause of morbidity and mortality, particularly in those who are obese.

The symptoms associated with organ fibrosis and loss of function are often found in more common diseases. Therefore, fibrotic disease is diagnosed following the exclusion of other illnesses. This was the case for patient “MH” who was diagnosed with IPF when he was 59. He first noticed a sudden shortness of breath when he was at elevated altitude, which previously he had ignored due to their infrequent nature in the earlier stages of the disease. Many things can cause breathlessness, such as asthma, allergies, respiratory infections, and therefore we all experience this “common symptom of IPF.” However, it was only when he was at altitude and the symptom persisted, that he sought medical advice. He was initially diagnosed with cardiac insufficiency in addition to his underlying asthma, therefore he was given a stent and an inhaler. When his symptoms didn't significantly improve he was then referred to a pulmonologist, who diagnosed IPF. Due to the rare nature of this condition it is perhaps not unsurprising that it takes time to make a formal diagnosis of IPF, as one presumes that the treating physicians are more likely to try and exclude other common diseases where therapeutic options are more readily available before presenting the diagnosis of IPF to a patient. Likewise, patient “KF's” condition of systemic sclerosis also took 6 months before being diagnosed with her disease. Whilst the priority of research should be on the discovery of new therapeutic treatments, the thoughts raised from patients also highlight the need for better tools and biomarkers to provide a more rapid diagnosis of disease. Certainly, now that pirfenidone and nintedanib have both been identified and approved for the treatment of IPF, one presumes that treating patients in the early stages of diseases would be preferential.

KF was ~30 years old when she first noticed persistent pins and needles, typically associated with Raynaud's disease, a condition affecting up to 20% of females. In KF's case, her physician sent her for follow up tests and was diagnosed with systemic sclerosis (SSc). The challenge with diagnosing conditions such as systemic sclerosis is that many diseases can present in a similar way, such as mixed connective tissue diseases with features of rheumatoid arthritis or systemic lupus erythematosus, or even diseases like amyloidosis and paraneoplastic syndromes. Recognizing the importance of improvements in diagnosis, a new set of classification criteria recently been proposed (van den Hoogen et al., 2013). Nevertheless, these new criteria are complex and very subjective with the only objective measure being the presence of SSc-related autoantibodies. In the 6 months that it took to diagnose KF, her ability to perform everyday tasks became significantly worse such as brushing her hair or picking things off the floor. This rapid deterioration is not uncommon and in recognition of this the EUSTAR^1^ group has undertaken a study to define preliminary criteria for the very early diagnosis of systemic sclerosis (VEDOSS). Based on this study, early features are the presence of anti-nuclear antibodies, SSc-specific antibodies, SSc pattern on nailfold capillaroscopy and puffy fingers in Raynaud syndrome patients (Minier et al., 2014). Unfortunately even with early diagnosis there is currently no cure for SSc, however with increased monitoring the opportunity to manage systems and to reduce the impact of some of the more severe complications associated with this disease, such as pulmonary arterial hypertension and renal crisis, should be improved. Hope does remain that therapies are being developed that might tackle this disease and it is noteworthy, and illustrates the eagerness of regulatory authorities to bring medicines forward, that the FDA recently gave Roches Actemra (tocilizumab), an anti-IL-6 receptor antibody, breakthrough status for systemic sclerosis^1^ even though the primary endpoints weren't met, but showed a positive trend in Rodnin skin score, in their recent Phase II study (Khanna et al., 2014).

Due to the diffuse nature of the fibrosis in SSc options for transplanting affected organs are not possible. In the case of IPF and autoimmune hepatitis, lung, or liver transplant remain an option, but naturally this is often the last available option. Patient ADL was only 17 when she was first diagnosed with autoimmune hepatitis (AIH) after she observed a yellowing of her eyes, a common diagnostic feature of liver injury and damage. Like the previous described conditions the actual cause of this fibrotic condition is unknown and due to the rare nature of this condition diagnosis is more about excluding other autoimmune conditions, such as thyroid disease or pernicious anemia in as much as confirming the disease. Unlike IPF, autoimmune diseases such as SSc and AIH, are both treated with immunosuppressants, such as oral steroids and azathioprine, with often limited success. Certainly, in ADL's case this seemed to be the case with her noting that “nothing seemed to help the inflammation.” Tragically, the therapies provided often put the patients at increased risk of infection. On her 20th birthday, ADL was admitted to the intensive care unit with infected abdominal ascites fluid from the scarred liver. Unfortunately, for AIH patients the only therapeutic option for managing the disease is maintaining patients on a life time of immunosuppressants balancing inflammation versus the risk of infection, consequently patients are regularly monitored and physicians are constantly looking to taper down high dose steroids and manage their patients with antibiotics. With little change in treatment course or prognosis this can often leave many patients with a fibrotic condition with a sense of loss, frustration, and depression about their lives.

As can be seen in all of the patient perspectives, there is significant morbidity associated with tissue fibrosis and also a relatively poor prognosis, due to the lack of anti-fibrotic therapies. Early diagnosis in many chronic diseases is known to result in better outcomes. Detection of type 2 diabetes close to disease onset can result in diet controlled glucose regulation and the lack of need for medication; early cancer diagnosis can allow for surgery and removal of tumors; early changes in intraocular pressure can prevent glaucoma and preserve eyesight. Hopefully, as our understanding of diseases associated with tissue fibrosis increases, we may find more patients being diagnosed earlier in their disease course, rather than diagnosing these conditions as a matter of exclusion of other more common illnesses. Moreover, the number of GWAS studies assessing risk factors for tissue fibrosis may identify susceptibility markers that can identify patients either early in their disease, or help prevent fibrosis from ever occurring (Radstake et al., 2010; Anstee and Day, 2013; Fingerlin et al., 2013; Noth et al., 2013; Tampe and Zeisberg, 2014; Zain et al., 2014).

Also, as we begin to dissect the causes of fibrosis, we will be in a stronger position to prevent fibrosis thus retaining tissue function. Liver fibrosis associated with hepatitis C infection is a good example of this. Anti-virals have been reported to inhibit hepatic fibrosis (Shiffman and Benhamou, 2015), but this is not due to a direct effect of the compounds on portal fibroblasts or collagen secretion, but actually due to the fibrosis being deposited in the liver as a consequence of hepatitis C. Therefore, if you remove the viral trigger, then the liver can repair itself. Another example is the kidney, where high blood glucose is known to be another fibrotic trigger as nephropathy is observed in diabetics with poorly managed blood glucose. The triggers are less clear in the lung, with many different factors being postulated such as smoking or other environmental stimuli, radiation for cancer therapies or certain medications (Oh et al., 2012). However, all of these triggers have been observed in small groups of patients.

Currently, the medical and pharmaceutical community are trying to find therapeutic options that can stop the progression of disease and a number of these pathways are being tested in clinical trials in different organs simultaneously, such as anti-LOXL2, LPA1 inhibition or galectin-3 inhibition in lung, liver, cardiac, and systemic fibrosis. Future approaches may also be able to promote a resolution of fibrosis and ultimately repair the damaged tissue. The liver regenerates after hepatitis C eradication. The ability of the lung to regenerate is controversial. However, in the acute setting of ARDS, patients have diffuse alveolar damage, which exhibits many histologic and radiographic features of IPF, and can go on to resolve the fibrotic phenotypic changes (Lynch, 2001; Nöbauer-Huhmann et al., 2001; Burnham et al., 2014), therefore indicating that lung repair is possible.

What is striking about all fibrotic diseases is the loss of dignity that many of these patients must feel, a sense of burden on their family, an inability to take on common daily tasks such as brushing your hair, and relying on help for basic bodily functions. In addition all of these diseases leave the patients with disability which makes them increasingly withdraw from society and the broader family network. It is clear though that a close relationship is often established with the treating physician and in many cases the willingness of the patient to enroll and be involved in experimental drug trials such as those being run by the IPFnet, or to support patient support groups or charities for their respective condition such as the Scleroderma Society or Pulmonary Fibrosis Foundation. These support groups highlight a common feature across fibrosis: the motivation of patients and physicians to help advance our understanding of disease and ultimately be able to provide therapeutic options to patients with these devastating conditions.

## Conflict of interest statement

The authors declare that the research was conducted in the absence of any commercial or financial relationships that could be construed as a potential conflict of interest.

## Footnotes

^1^http://www.gene.com/media/press-releases/14596/2015-06-10/fda-grants-breakthrough-therapy-designat.
