# Supplementary material for: Living with Fibrosis: From Diagnosis to Future Hope
Source: Front Pharmacol. 2015 Dec 16;6:288. doi: 10.3389/fphar.2015.00288 (PMC4684076; doi:10.3389/fphar.2015.00288)
Supplement: Supplementary file 1 [file DataSheet1.DOCX]

My journey through idiopathic pulmonary fibrosis

Michael C. Henderson1, Daniel M. Rose, MD1

1 The Pulmonary Fibrosis Foundation, Chicago, IL, USA

Imagine being told that you had a disease you had never heard of, with no known cause, for which there was no treatment and no cure. Your future had been instantly shortened to 3 to 5 years. I’d like to share with you the story of how I was diagnosed with IPF, the progression of the disease and its impact on me, the difficult decisions I had to make along the way, and how I’m working to help others that have been afflicted with this disease. Here is how things unfolded.

**My symptoms before diagnosis**

In October 2003 I was at a conference at Squaw Valley, elevation 6500 feet. I took a break for my daily walk and found myself gasping for air. For the rest of the meeting I found myself stopping to catch my breath any time I exerted myself. This was definitely not normal for me. At home in Portland, Oregon, I was walking a few miles a day on a regular basis without shortness of breath.

I knew something was wrong. Looking back, I recall similar episodes dating back 3 years where I had comparable symptoms at elevation while skiing or hiking. My doctors attributed it to coronary artery disease or asthma. So, with a new stent and an inhaler, I began cardiac rehab in January 2004.

**My diagnosis**

I thank God for a cardiac rehab nurse who pulled me aside after a few weeks and said, “Your oxygen saturation is dropping too low and we don’t think it is asthma or cardiac. You really should see a pulmonologist.”

In June my pulmonary physician diagnosed me with IPF. He said there was no effective treatment and no cure, and that I should get my affairs in order. He told me that I had no more than 3 to 5 years to live. That’s it. That was the message. Talk about a shock -- try telling that to your wife and family! My whole world turned upside down that day. I was 59 years old, looking forward to retiring, rafting, fishing, hiking, traveling, and having more time to spend with my kids and grandchildren. Many of the things I loved to do suddenly looked out of reach. Why me? Why now? What caused this? What do I do now?

**My progression**

I felt sorry for myself for a day or two, and then I focused on learning about the disease and what I could do. Thanks to the Internet, I learned that there were clinical trials available and that I should get on a lung transplant list. I learned that one of the most knowledgeable IPF doctors in the world, Dr. Ganesh Raghu, was in my backyard in Seattle. I went to work trying to get an appointment to see him.

That took a while, so my wife Donna, and I decided to test how I could manage at elevation. In August 2004 we drove to Hurricane Ridge in the Olympic Mountains, about 5000 feet. I could not walk across the parking lot without stopping to catch my breath. I tried walking up a short trail that had a slight grade. I failed that test. In November we decided to knock a few things off my bucket list and took a road trip that included Bryce and Zion National Parks in Utah. We didn’t even think about the elevation. It was spectacular, but I had to view some of it through Donna’s eyes. If it was a long walk or uphill to see the sights, she went, came back, and told me how spectacular it was. It’s a good thing I’m not one who gets depressed easily. That could have done it. I was no longer in control. By the time I saw Dr. Raghu in December 2004 my shortness of breath at sea level was not too bad but was slowly getting worse. By March 2005, I needed oxygen at night and for exercise, which helped greatly. I bought a pulse oximeter to manage my exercise. By April, my deterioration began to accelerate. Without oxygen, a slight grade became difficult and stairs were a killer. I put together a backpack of oxygen and continued to exercise almost daily at a cardiac rehab program. With 2 liters of oxygen I was able to walk a few miles. I felt good physically. I even took a fishing trip in May with my brother-in-law. We took 42 tanks of oxygen and used them all.

The symptoms were not what I expected. Shortness of breath seemed to be a real problem with quick movements, like getting up to walk across the room. If I moved slowly at first, I didn’t get short of breath. If I moved quickly, I did. I could feel the deterioration in lung function. For me it was a stair-step effect. It might be a few months or only a few weeks, but the decrease was noticeable. Sure enough, the pulmonary lab would confirm the impairment.

**What about the cough?**

In July 2004 I went on a fishing trip in Canada. It’s my first memory that cough was becoming an issue. I remember being yelled at by one member of the group - a first class “jerk.” He was very irritated by the continuous clearing of my throat. It happened about every 30 seconds. Good thing we didn’t share a tent. For some people, the cough is the worst part. It is absolutely debilitating. I never had it that bad. It was more a constant clearing of my throat, just plain irritating to me and to those around me. I only coughed when speaking or out of breath.

**Treatment options**

I did a lot of homework on this issue. The most interesting possibilities to me were Gleevac, Actimune and pirfenidone. I spoke with Dr. Brian Drucker at University of Oregon Health Sciences Center about Gleevac. It required a move to Minnesota to be at the Mayo Clinic. I saw the data on the Japanese pirfenidone trial and was very enthused. Imagine: a pill that might stop the disease from progressing. But, it was unavailable in the US. The University of Washington was conducting a clinical trial of Actimune, which had shown some promise. A friend suggested I visit John of God in Brazil. I was desperate. I would have done anything, gone almost anywhere to find a cure. In March 2005, Dr. Raghu and I discussed the only practical treatment options: do nothing, try the IPF cocktail of prednisone, azathioprine, and NAC (n-acetyl cysteine), or participate in a clinical trial. The only promising trial available locally was interferon gamma 1b (Actimune), and Dr. Raghu strongly encouraged me to participate. My research told me that it was also available off label so I asked him to consider that as an option. He laid out the pros and cons of all the options and left the choice to me. So after careful thought and some more research, I chose a course of prednisone, NAC and Actimune. I wanted to make sure I was actually getting Actimune. I understood the value of the clinical trial, but if I could know that I was actually on the drug, I would find some other way to give back to the research effort. Of course the Actimune trial was terminated shortly after I made my decision, so we went to the IPF cocktail (prednisone, azathioprine and NAC).

**Continued disease progression**

By the fall of 2005, speaking became more difficult. My voice was softer and I coughed frequently. I could still walk slowly around town with 2 liters, but everything became more difficult. Showers were the worst. They were exhausting. Bending over to tie my shoes became a challenge. I became more sedentary. I still went to work every day but put in less time. By December I was on oxygen 24/7, otherwise I would be out of breath before I reached the bathroom.

**How IPF changed my life**

When I look back I realize that IPF changed everything. Physically you walk instead of run; you avoid hills and stairs; you don’t feel like exercising; you tire easily; and you lose your appetite. You can’t do the things you want to do: fishing, hiking, and traveling. You end up in a wheelchair. Your life is changed socially, you avoid crowds and parties, and your friends go to fun places without you. You become self-conscious, your cough irritates those around you, and the sound of the oxygen concentrator irritates people nearby. People shy away from you, they are afraid of what you have. One of my IPF friends had an incident in an elevator. He said, “Don’t worry it’s not contagious, it’s just terminal.” The impact on my family was profound. My spouse was stressed-out and my kids were unsure how to respond.

The mental aspects are quite difficult. At times I became depressed, which was very unlike me. I was angry. I got tired of answering “How are you feeling?” When one can’t breathe, one can’t think. I became fearful: will I be able to keep my job? Will I run out of oxygen? Can I fly? Can I go on a cruise? One begins to wonder: will my sibling or kids get IPF? What is it like at the end?

**The lung transplant**

I made up my mind early to pursue a lung transplant. If I could have a 50/50 chance of having a good quality of life for 5 years, I would do it. My biggest fear was not death but continued debilitating medical issues. I was listed for transplant on December 28, 2005. I received two new lungs on March 19, 2006. What an amazing, incredible gift. There were some serious bumps in the road but I came through fine.

**Condition today**

So, how am I doing now? I have to say much better than I expected. My cough is gone. I am only short of breath when climbing stairs or walking a steep grade, especially at high elevation. I can actually run a fair distance. I am leading a pretty normal life. I still work about half time. I am able to travel to “safe” places.

I exercise about 4 days a week. I fish again. I lead two IPF support groups and a lung transplant support group. I joined the Board of the Pulmonary Fibrosis Foundation. I’m still working to pay back for choosing “off-label” over the clinical trial.

Today my lungs may be the best part of me. I celebrated my 5th “Transplantiversary” on March 19, 2011 with a huge party for all my friends and family. I’m already planning for the 10th “Transplantiversary” in 2016. I am one of the lucky ones. The way I calculate, less than 2% of IPF patients are able to have a lung transplant. Realistically, I shouldn’t be here today.

**My Involvement with the Pulmonary Fibrosis Foundation**

At the suggestion of my pulmonary physician, Dr. Raghu, I became involved with the Pulmonary Fibrosis Foundation (PFF). I joined the Board of Directors in November 2011 and subsequently became Vice-Chairman of the Board in November 2013. I also serve on the Executive, Finance, and Governance Committees.

The Foundation is involved in many activities to help improve the quality of life for the patients, family members, and caregivers impacted by pulmonary fibrosis. Presently they are beginning to implement the PFF Patient Registry and the PFF Care Center Network that will standardize and improve the care patients receive. Additionally, the Network should increase the efficiency for patient enrollment in clinical trials. Finally, they will attempt to coordinate multi-center research through public and private partnerships.

Furthermore, the Foundation hosts and supports a number of educational activities. They recently held their biannual international conference PFF Summit: From Bench to Bedside in La Jolla,

CA in December 2013. This event was attended by over 500 individuals and included patients, caregivers, healthcare professionals, industry and government representatives, and members of the financial community. The Foundation has assisted in the presentation of local educational events as well as similar events in the EU.

Finally, the PFF is closely involved with expansion of the patient support group network including both in-person and on-line meetings. They have held support group leader workshops in the US and EU, and are creating a US and International Patient Advisory Council to enhance the communication between the patient community and the Foundation.

I have been involved in the planning and implementation of many of these activities. They have been personally rewarding and, even more importantly, a way to give back to those who have so generously supported me. As an active member of the IPF and transplant communities, it seems that I am contacted almost weekly by newly diagnosed patients and their families. It is such an honor to share my experience with others, and I hope that my success story makes them feel less alone and more hopeful. And while the hardest part of my involvement is attending memorials of friends, their passing also highlights the importance of my work with the PFF and the urgent, critical need for a cure.

For more information on the Pulmonary Fibrosis Foundation, visit

<http://www.pulmonaryfibrosis.org>.
