# Supplementary material for: Living with Fibrosis: From Diagnosis to Future Hope
Source: Front Pharmacol. 2015 Dec 16;6:288. doi: 10.3389/fphar.2015.00288 (PMC4684076; doi:10.3389/fphar.2015.00288)
Supplement: Supplementary file 2 [file DataSheet2.DOC]

Living with systemic sclerosis

Kim Fligelstone

I was a happy 28/9 year old having just bought a large converted Victorian house with my boyfriend which needed extensive work to restore back into a house. We both had busy jobs so almost every spare minute was taken up with paint stripping (me) and building work (Paul), and the occasional gig (I love live music even now some 26 years on). So when I started to feel tired it was put down to general over working and somewhere in the back of my mind I thought it may be aging after all I was approaching the big **30**. One evening I was moaning that I couldn’t put any weight on my elbows when turning over because of the pain and kept waking up with pins and needles and sometimes one of my arms or hands would feel numb in the middle of the night. At this point I went to my GP who told me I had a very common condition called Raynaud’s (bad circulation) and asked if my hands changed colour in cold weather. Actually I hadn’t noticed but sure enough when I looked at them they did. He was a very astute Dr and sent me for various tests including lung function, and to both rheumatology and dermatology specialists at the local Hospital, it took about 6 months (very quick) using a process of elimination to diagnose diffuse systemic sclerosis, this is a rare connective tissue disease which can affect the skin, gastrointestinal tract, lungs, heart and kidneys as well as soft tissue. Fortunately for me I lived in London and a World scleroderma specialist Prof Carol Black was at the West Middx Hospital so I was referred immediately. By this time I was feeling pretty unwell taking up to 45 mins to walk from the tube to work which would usually take 10 mins, climbing stairs on my hands and knees, my fingers were swollen which meant I couldn’t do much if any DIY. I was coming back from work completely exhausted going straight to bed then getting up for work the next day, I seemed to be going downhill very quickly. The specialist arranged for me to spend some time in Hospital to undergo a series of tests when I overheard one of the nurses saying “poor girl she hasn’t a clue what’s wrong with her”, to be honest I knew they were talking about me but I fervently hoped I was wrong. There was very little information available for people with scleroderma in those days and of course the Internet wasn’t around which was a mixed blessing; there can be too much misinformation which can scare people today. I had been told that I was very sick and although there wasn’t a cure, there were several experimental treatments to try to stop the progression. Around about this time I realised I needed to talk to someone other than my partner, family and friends, I was terrified about what the future might have in store and didn’t want to scare anyone so I went to see a therapist, who knew nothing about the disease but could see that I was in trouble. Of course I’m relaying this story with hindsight the most difficult thing for me at the time was fear of the unknown and as I deteriorated my mobility became really affected, I could no longer bend over to pick something off the floor and I couldn’t raise my arms far enough to brush or wash my hair, once I fell over and couldn’t get up without kind people to help. My skin had become tight all over my body and the same process was happening to my tendons so having a bath, stairs was either impossible and or painful. It’s incredible what we take for granted until we face a challenge or obstacle, fortunately there are now aids to help in everyday life, then we had to be inventive for example buying tubing for cutlery handles, a home made back scratcher made out of a hair brush and metal clothes hanger to scratch my back which was incredibly useful as I was very itchy in the early part of the disease. Probably my most useful gadgets were a pick up stick and aid to help put on socks. On the positive side (for me) I stopped all forms of house work and learnt to drive to make sure that I wasn’t going to be housebound or totally dependant on my partner.

Not well enough to go back to work I took up a part time university access course at the local college and went on the inevitable rounds of various hospitals for tests and treatment.

In the winter of 1991on my way back from lung function tests at the Brompton Hospital I remember getting stuck in the snow; I’m not sure how long it took me to get home around 4 hours and I went straight to bed with an excruciating headache. I came round 3 days later having gone into a coma in the early hours of the morning being rushed to the local hospital, where I stayed on a trolley in the corridor for 24 hours (not enough beds) and had 3 grand mals (fits) then transferred to the Royal Free Hospital in Hampstead where Professor Black was now running the 1st specialist scleroderma department in the UK. My partner was told that they threw everything they could at me and to prepare for the possibility of brain damage or worse then luckily for me I woke up, angry (probably from the pain) told a junior Dr who is now my consultant to f….o and was in a pretty confused state. For 2 weeks I was forced to drink as much liquid as possible to try and kick start my kidney’s into working again. This didn’t happen so I started haemodialysis where the blood is removed by machine, cleansed of toxins before re-entering and circulating the body. I felt as though everything was in slow motion and I was probably in shock from kidney failure anyway I lost half my body weight continually throwing up eventually had a feeding tube fitted and started on Peritoneal dialysis which uses the peritoneum (membrane that lines most of our internal organs) with a glucose solution to remove toxins. My body adapted well to this, although it took my mind a bit longer to adapt and I continued to improve so much so that my kidney function was good enough to stop dialysing 2 years afterwards but my scleroderma was also improving, my skin and mobility were much better, so I continued to dialyse until 2008 and only stopped because masses were found on my ovaries during an ultra sound of my peritoneum. The silver lining in this case was that the silent killer had been found early enough to be removed before spreading. One tumour was borderline the other malignant and 2 operations and 5 years later I have been discharged from UCH gyny oncology department. Hooray!

The above account really only touches on the beginning of the disease and major life threatening aspects of my living with scleroderma annoyingly there are others such as soft tissue loss, resulting in loosing fatty pads on soles of my feet, causing a change in appearance with a very prominent nose as the skin tightens and the same process makes my fingers look very slim (albeit bent from years of tight skin) and calcium deposits called calcinosis which can come out of the skin causing enormous pain and lead to ulceration. I have annual tests for Gastro dysfunction, mild lung fibrosis, Barrett’s (precancerous cells in oesophagus) due to reflux caused by scleroderma and eosinophilic colitis, who knows what caused that. Thankfully so far annual tests for pulmonary hypertension and cardiac involvement are clear.

Throughout my life I have tried, reasonably successfully, I think to have not let scleroderma or any illness stop me from living my life to the best of my ability. Ok I watch a lot of bad TV but that’s my way of resting. Not long after starting peritoneal dialysis my partner and I visited friends in Australia, we had to be more organised than usual and our friends’ houses sometimes looked like a dialysis centre but I felt that I was doing normal things. When I was able to dialyse less I took holidays for a maximum of 2 weeks at a time and travelled to Europe, Thailand and China. Last year I had a blip and was in hospital for 2 weeks with a fairly long recovery time which meant I had to cancel several work related trips but promised myself that if everything turned out ok I would do something else and an opportunity came up to spend Christmas and New Year in Vietnam and against all the odds I went and was virtually the healthiest person on the trip! Just to prove that it hasn’t all been one big holiday I am a volunteer with the Scleroderma Society, having been Chair from 2000 -2013, I took on the role of President of FESCA the European Scleroderma Associations Umbrella Federation from, 2006-2008. I work part time at the Royal Free Hospital visiting people on the ward, helping to organise local meetings and admin work. I’m on several helplines (no one needs to know you’re in bed if you’re on the phone) and over the years have been part of task forces and steering groups for scleroderma guidelines and recommendations.
