# Supplementary material for: Living with Fibrosis: From Diagnosis to Future Hope
Source: Front Pharmacol. 2015 Dec 16;6:288. doi: 10.3389/fphar.2015.00288 (PMC4684076; doi:10.3389/fphar.2015.00288)
Supplement: Supplementary file 3 [file DataSheet3.DOCX]

**Liver Cirrhosis – A patient’s perspective**

Angela DeLisle

Clinical Instructor, Section of Digestive Diseases, Yale University 333 Cedar Street, 1080 LMP, P.O. Box 208019, New Haven, CT 06520-8019 Tel: 203-737-6063,

E-mail: ANGELA.DELISLE@ynhh.org

The author has no conflicts of interest

*“Wondering what tomorrow will bring”.*  That’s my status update today. I try to keep it light these days. Nobody really wants to hear the truth: “*Wondering if I will bleed again?”,* “*Wondering if my ascites is infected again”, “Wondering if I will live to see my 25^th^ birthday…” .*  I put the laptop down to rest. I will keep this status for now. Thinking of an alternative is simply too exhausting.

I haven’t always been this melancholy. I was once vibrant, happy …. healthy. I am 22 years old with cirrhosis from autoimmune hepatitis. Up until I was 17, I was your typical immortal teenager. I laughed with my friends, loved to shop, was even enrolled in Community College. Then, one day, I noticed that my eyes appeared yellow. My doctor did some tests and referred me to a liver doctor. After more tests and even a liver biopsy, I was told that I had an “autoimmune process” in my liver – autoimmune hepatitis with some “scarring” on my liver. I was placed on a few regimens over the years, but nothing seemed to help the inflammation.

On my 20th birthday, when I had plans to celebrate with my friends, I awoke feeling nauseated. I vomited that day. Blood. Lots of blood. I don’t remember much else about that day. My mom filled me in on the days that followed. I was in the Intensive Care Unit, a breathing tube helped me to breath. I had a few endoscopies where they placed rubber bands on veins in my throat that were bleeding. Finally the bleeding stopped. I was also told that I had “fluid in my abdomen” – ascites – and that this fluid is now infected. The doctors who took care of me during this hospital stay told me that this sometimes happens with a variceal Bleed.

After my hospitalization, I had to visit my liver doctor frequently to undergo more endoscopies with banding of the remaining varices. He said that I would have to continue to have the endoscopies until all the varices are gone. In addition, I have to take a medicine to lower my pulse. He explained that by lowering my pulse, we are also lowering the pressure within the liver and may prevent a repeat variceal bleed. A side effect of the medication is that I constantly feel tired. Sometimes I get dizzy when I stand up so I try to have someone with me during those times.

That hospitalization marked a turning point in my liver disease. After my variceal bleed, my liver doctor referred me to a Liver Transplant Center. I went through the rigorous evaluation and was placed on a national Wait List for those waiting for a liver offer - “Listed”. That day I felt hope for the first time in years. My brother and a cousin later came forward to be evaluated as living donors but neither were a “fit”. My prior hope then waned….

Over the years I have become familiar – too familiar- with terms such as thrombocytopenia, ascites, varices, jaundice and encephalopathy. I learned about that last one only recently. One day I couldn’t remember words, forgot the access code to my phone and didn’t recognize my brother. I lost it emotionally. My mom brought me to the Emergency Department where we were met by my liver doctor. I was diagnosed with hepatic encephalopathy and now have the pleasure of taking pills and a sickly sweet syrup – lactulose – a few times a day. This makes me move my bowels a lot and clears out toxins. It was difficult in the beginning to know how much or how little to take. I soiled myself a few times when I took too much. Because I am so weak, I am unable to clean myself and have to rely on my visiting nurse or my mom to help me. What 22 year old should have to bear such disgrace ??

Another change over time has been my nutrition. I have lost weight over the last few years. Most 20-somethings would be thrilled to lose weight without trying. I am not. My arms and legs are skeletal and my abdomen is round with ascites. I feel like my appearance would frighten children if I had the energy to leave the house. I am told to eat; that nutrition is as important as my other medicines. Easier said than done. I am too exhausted to eat and the size of my abdomen makes me feel perpetually full.

Ascites has also become an issue. Early on, my liver doctor spent a great deal of time explaining why it developed – something about increasing pressures in the liver ….. I think my explanation is better: the liver is crying because it knows that it’s failing. Either way, I am resigned to taking water pills. I am told they work in different manners but work better together. The result is that I am always in the bathroom urinating. Most days I do OK with the regimen, some days I have accidents. It is humiliating and embarrassing. Because I had the infection in my ascites at the time of my hospitalization, I now have to take a daily antibiotic to ward off future infections. What they don’t tell you right away is that by taking the antibiotic, it will prevent certain bacteria from infecting the ascites but may predispose you to stronger, more resistant bacteria down the line. My liver doctor says that I should take the antibiotic for two reasons: because of my prior infection and because the protein in my ascites is very low – whatever that means. I guess the options of taking the antibiotic or not taking the antibiotic come down to this : risking a repeat bout of peritonitis versus risking the development of a super-bug. It doesn’t look like I have great options here. So I take the antibiotic and cross my fingers.

Since being listed two years ago, I have been waiting for a Liver Offer. It has been a long two years. I feel like the diagnosis of my liver disease, more specifically, the diagnosis of cirrhosis, has stolen my youth. I used to be Field Hockey captain. Then, in my senior year, I was sidelined because my spleen was too big to safely play. I never went to Prom. I was too embarrassed to be seen in a gown with the changes that happened to me from my steroids – I was puffy with acne. Who wants pictures to memorialize that ? I never finished college. The fatigue and joint pains prevented me from focusing on my studies. I slowly lost friends. I can understand that no-one wants to be around someone who is constantly sick, tired, confused or angry. Yes, I can understand but it doesn’t make it any easier when it happens. I still have a few close friends who I am thankful for. I used to be bitter and resentful regarding my cirrhosis but now I have accepted that this is how things are going to be. I would like to think that I have matured and my acceptance is a reflection of that but the reality is that I am now too tired to fight. Or care.

I am suddenly awoken by the sound of movement. In my drowsy state, I vaguely hear the phone ring and then not so vaguely hear my mom scream in excitement. Breathless, she rushes back to me to tell me the good news: There is a liver offer. I look at my laptop resting by my side and see my last Status Update : *Wondering what tomorrow will bring*. I choose to leave it. This time, instead of thinly-veiled cynicism, I feel genuine hope, a prospect for the future. *Wondering what tomorrow will bring* ? Yes, Indeed. I certainly do wonder……..
